# Correction: Progression of carotid intima-media thickness, visceral fat accumulation and metabolic derangement in people living with HIV initiating antiretroviral therapy: A prospective cohort study at Thailand’s tertiary care center

**DOI:** 10.1371/journal.pone.0345774

**Published:** 2026-03-24

**Authors:** Thirajit Boonsaen, Winai Ratanasuwan, Boonrat Tassaneetrithep, Tanyaluck Thientunyakit, Jitsupa Wongsripuemtet, Shanigarn Thiravit, Phatharajit Phatharodom, Oranich Navanukroh, Mayuree Homsanit

In the subsection titled “Study participants” within the Methods section, there is an error in the fourth sentence of the first paragraph. The correct sentence is: The study protocol was approved by the Siriraj Institutional Review Board (SIRB), Faculty of Medicine Siriraj Hospital, Mahidol University (COA no. SI341/2016, approval date 31 May 2016; renewed annually through 30 May 2021).
