# Supplementary material for: Progression of carotid intima-media thickness, visceral fat accumulation and metabolic derangement in people living with HIV initiating antiretroviral therapy: A prospective cohort study at Thailand’s tertiary care center
Source: PLoS One. 2025 Oct 30;20(10):e0335242. doi: 10.1371/journal.pone.0335242 (PMC12574832; doi:10.1371/journal.pone.0335242)
Supplement: Attachment — Submitted filename: Response to reviewers.docx [file pone.0335242.s011.docx]

# **Manuscript title:** **Progression of carotid intima-media thickness, visceral fat accumulation and metabolic derangement in People living with HIV Initiating Antiretroviral Therapy: a prospective cohort study at Thailand’s tertiary care center**

**Journal:** PLOS ONE

**Manuscript ID:** [PONE-D-25-27072] -[EMID:c21e87286d82c702]

We thank the Academic Editor and both Reviewers for their thoughtful and constructive feedback. We have carefully revised the manuscript in response. Below we provide a point-by-point response, with reviewer comments in *italics* and our replies underneath. All changes have been incorporated into the revised manuscript, which includes a tracked-changes version as requested.

## Reviewer #1

**1. Abstract – Use of abbreviations (“cART”, “PLHIV”).**

*Comment: Align with international guidelines, use “ART” and “PWHIV.”*

**Response:** We have revised the terminology throughout the abstract and main text, replacing “cART” with “ART” and “PLHIV” with “PWHIV.”

**Revision:** Abstract, p.1; throughout the manuscript.

**2. Abstract – Clarity of “metabolic dysregulation / metabolic parameters.”**

*Comment: Please specify what is meant by “metabolic dysregulation/parameters.”*

**Response:** We clarified this phrasing to specify fasting glucose, HOMA-β, lipid profile, and renal function as metabolic parameters, and replaced “metabolic dysregulation” with “early metabolic alterations.”

**Revision:** Abstract Results/Conclusions; Introduction p.2.

**3. Abstract – Inconsistent reporting of % change and p-values.**

*Comment: Please standardize expression of results.*

**Response:** We have standardized the reporting of results in the Abstract and Results sections, presenting mean change with % change and p-values consistently.

**Revision:** Abstract Results; Results section, Tables 3–4.

**4. Abstract – Conclusions overstated.**

*Comment: Proactive surveillance not supported by data.*

**Response:** We rephrased the conclusion to state: “These findings underscore the need for monitoring cardiometabolic risk in PWHIV on ART,” without implying cost-effectiveness or clinical recommendation beyond our data.

**Revision:** Abstract Conclusions, p.1.

**5. Introduction – Lipodystrophy and outdated ARVs.**

*Comment: Intro emphasizes older ART toxicity not relevant today.*

**Response:** We revised the introduction to distinguish between historical lipodystrophy and modern ART, clarifying that while lipodystrophy is uncommon with current regimens, visceral fat accumulation and altered fat distribution remain concerns. Additional references were added.

**Revision:** Introduction, p.2.

**6. Aim of study unclear.**

*Comment: Clarify study aim at end of introduction.*

**Response:** We revised the final paragraph of the Introduction to clearly state: “This study aimed to characterize 36-month longitudinal changes in fat distribution, metabolic parameters, and carotid intima-media thickness (cIMT) following ART initiation in Thai adults.”

**Revision:** Introduction, last paragraph.

**7. Language – people-first.**

*Comment: Avoid “HIV-infected”; use “people living with HIV.”*

**Response:** Revised throughout manuscript.

**Revision:** Global changes.

**8. Methods – Generic “metabolic changes.”**

*Comment: Specify exact variables, primary vs secondary outcomes.*

**Response:** We revised Methods to specify primary outcomes (changes in fat distribution and cIMT) and secondary outcomes (changes in glycemia, lipids, renal indices).

**Revision:** Methods, Study design, p.3.

**9. Methods – Heterogeneous population (advanced vs recent HIV).**

*Comment: Consider stratification or sensitivity analysis.*

**Response:** We acknowledge the reviewer’s suggestion. However, due to the modest sample size, we did not perform stratified or sensitivity analyses by baseline CD4 level or duration of infection. Subdividing participants into smaller subgroups would have substantially reduced statistical power and limited the interpretability of the results. Instead, we presented overall cohort outcomes and discussed the potential influence of baseline heterogeneity as a limitation of the study.

**Revision:** Limitations section, p.27

**10. Methods – ART regimens not described.**

*Comment: Important confounder (e.g., PI, TDF, NNRTI).*

**Response:** We added Table 1 summarizing ART class exposure during follow-up and discussed class-specific effects in the Discussion.

**Revision:** Results Table 1; Discussion p.25-26

**11. Methods – Virological suppression not shown.**

*Comment: Necessary to demonstrate ART adherence.*

**Response:** We added data on viral load suppression and CD4 recovery (Table 2).

**Revision:** Results, Table 2

**12. Methods – Language (“patients” vs “participants”).**

**Response:** Revised consistently to “participants.”

**13. Methods – Sociodemographic characteristics missing in Results.**

**Response:** We agree that sociodemographic characteristics are valuable contextual data. However, these details were not systematically collected in our study and therefore could not be reported. We have acknowledged this as a limitation of the study.

**Revision:** Limitation, p.27-28.

**14. Methods – Statistical approach unclear.**

*Comment: Explain choice of tests and adjustment variables.*

**Response:** Expanded Methods: specified Wilcoxon/McNemar for baseline→12m changes; GLMM for longitudinal trends; covariates chosen a priori based on CVD risk factors.

**Revision:** Statistical analysis, p.8-9

**15. Results – Weight gain attribution.**

*Comment: Should not attribute solely to long-term ART; may be aging or return-to-health.*

**Response:** We revised interpretation in Results to neutrally report increases, and moved speculation about causes to the Discussion.

**Revision:** Discussion p.24-25

**16. Results – HOMA-β significance.**

*Comment: Borderline, should not be over-interpreted.*

**Response:** Rewritten to note only a transient decline at 24 months, with cautious interpretation in Discussion.

**Revision:** Discussion p.25

**17. Results – “Elevated adiposity” qualitative.**

**Response:** Removed qualitative phrasing; reported numeric data only.

**Revision:** Results, p.13.

**18. Results – Fasting glucose values not shown.**

**Response:** Added absolute values and change over time in Table 3.

**Revision:** Table 3

**9. Results – Renal function interpretation.**

*Comment: eGFR change may reflect transporter effects, not impairment.*

**Response:** Moved interpretation to Discussion; added note on potential ART–renal transporter effects (esp. TDF, INSTIs).

**Revision:** Discussion p.25-26.

**20. Results – Clarify whether correlations used baseline or change values.**

**Response:** Clarified that correlations were based on longitudinal changes at each timepoint, not baseline values.

**Revision:** Results/Table 5 legen

**21. Discussion – “Metabolic health” wording.**

**Response:** Changed to “metabolic parameters.”

**Revision:** Discussion p.24.

**22. Discussion – Clinical cut-offs of A/G ratio and cIMT missing.**

**Response:** Added references and cut-off values for adiposity (A/G ratio >1.0) and cIMT (>0.8 mm as subclinical atherosclerosis). Clarified that most participants remained below pathological thresholds.

**Revision:** Methods, Discussion p.8

**23. Discussion – HOMA-β interpretation overstated.**

**Response:** Rewritten to reflect only transient change at 24m, not persistent dysfunction.

**Revision:** Discussion p.14, 25

**24. Discussion – Weight gain may reflect “return-to-health.”**

**Response:** Added discussion of return-to-health phenomenon and baseline underweight subgroup.

**Revision:** Discussion p.24-25.

**25. Limitations – Not fully covered.**

**Response:** Expanded limitations to include regimen heterogeneity, lack of control group, possible ART transporter effects, manual IMT measurement, and confounding by unmeasured cardiometabolic drugs.

**Revision:** Limitations section, p.27.

**26. Conclusions – Overstated metabolic dysregulation.**

**Response:** Revised to conclude only: “long-term ART was associated with fat accumulation and progressive cIMT thickening, with modest early metabolic changes.”

**Revision:** Conclusions, p.28.

## Reviewer #2

**1. IMT measurement description incomplete.**

*Comment: Clarify CCA, bifurcation, ICA sites.*

**Response:** Methods revised to explicitly describe all sites measured, consistent with Table 4.

**Revision:** Methods, Carotid IMT measurement, p.8

**2. Operator blinding/variability not described.**

**Response:** Added detail: all scans were performed by trained radiologists blinded to clinical data; inter-reader reproducibility not formally assessed (limitation noted).

**Revision:** Methods, p.8; Limitations, p.27

**3. Manual vs automated IMT.**

*Comment: Manual measurement may not detect <0.1 mm changes reliably.*

**Response:** Acknowledged as major limitation in Discussion/Limitations.

**Revision:** Limitations, p.27.

**4. P-values reported as 1.000.**

*Comment: Unusual, clarify.*

**Response:** Clarified that p-values ≥0.99 were rounded to 1.000 following adjustment. Added explanation in Methods.

**Revision:** Methods/Statistics, p.9

**5. Preventive therapies (statins, antihypertensives) not reported.**

**Response:** Clarified that these were not systematically recorded, and acknowledged as limitation.

**Revision:** Methods, Limitations p. 27

**6. IMT correlation analyses – clarify value used.**

**Response:** Clarified that right carotid bifurcation mean values were used for primary correlation analyses.

**Revision:** Results/Table 5 legend p. 18, 21-22

## **Editorial Requirements**

- Financial disclosure: Revised to include “The funders had no role in study design, data collection and analysis, decision to publish, or preparation of the manuscript.”
- Ethics statement: Expanded with IRB full name, approval code, and written informed consent statement.
- Formatting: Revised manuscript to follow PLOS ONE style templates and LaTeX requirements.
- Terminology: Standardized throughout to ART, PWHIV, and participants.

All editorial requirements have been fully addressed, including the updated financial disclosure, expanded ethics statement, adherence to PLOS ONE formatting guidelines, and standardized terminology (ART, PWHIV, participants)
